# Safety and immunogenicity of the group B *streptococcus* vaccine AlpN in a placebo-controlled double-blind phase 1 trial

**DOI:** 10.1016/j.isci.2023.106261

**Published:** 2023-02-21

**Authors:** Majela Gonzalez-Miro, Andrzej Pawlowski, Janne Lehtonen, Duojia Cao, Sara Larsson, Michael Darsley, Geoff Kitson, Per B. Fischer, Bengt Johansson-Lindbom

**Affiliations:** 1 Immunology Section, Lund University, BMC D14, Lund, Sweden; 2 Minervax A/S, Ole Maaløes Vej 3, 2200 Copenhagen N, Denmark

**Keywords:** Immunology, Microbiology, Bacteriology

## Abstract

Group B streptococcus (GBS) is a leading cause of life-threatening neonatal infections and subsets of adverse pregnancy outcomes. Essentially all GBS strains possess one allele of the alpha-like protein (Alp) family. A maternal GBS vaccine, consisting of the fused N-terminal domains of the Alps αC and Rib (GBS-NN), was recently demonstrated to be safe and immunogenic in healthy adult women. To enhance antibody responses to all clinically relevant Alps, a second-generation vaccine has been developed (AlpN), also containing the N-terminal domain of Alp1 and the one shared by Alp2 and Alp3. In this study, the safety and immunogenicity of AlpN is assessed in a randomized, double-blind, placebo-controlled, and parallel-group phase I study, involving 60 healthy non-pregnant women. AlpN is well tolerated and elicits similarly robust and persistent antibody responses against all four Alp-N-terminal domains, resulting in enhanced opsonophagocytic killing of all Alp serotypes covered by the vaccine.

## Highlights

• Phase I study of a new maternal GBS alpha-like protein vaccine formulation (AlpN)
• The vaccine is well tolerated, with a good safety profile
• Vaccination elicits strong IgG1 responses to all clinically relevant Alp serotypes
• The IgG mediates opsonophagocytic killing of all serotypes covered by the vaccine

## Graphical abstract

**Figure:**
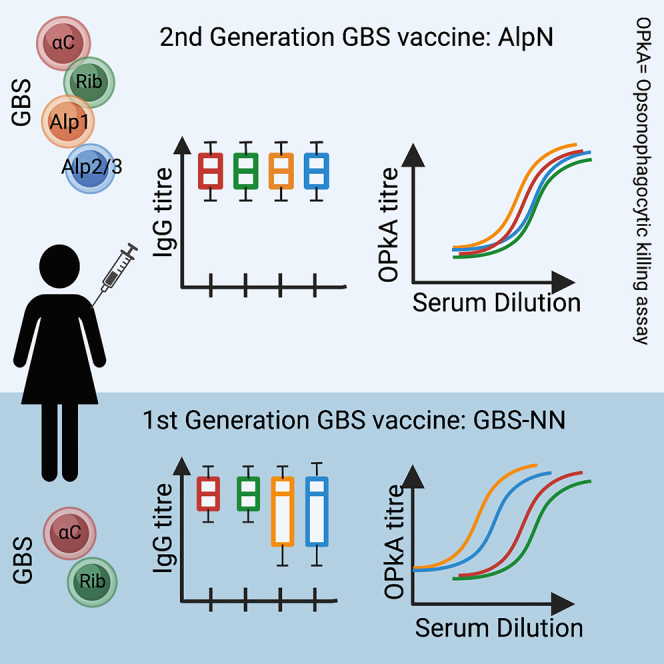

## Introduction

Group B streptococcus (GBS; *Streptococcus agalactiae*) is an encapsulated Gram-positive bacterium that colonizes the rectovaginal mucosa. It has been estimated that 18% of all women worldwide are colonized at any one time, but a clear regional variation in prevalence exists.^1^^,^^2^^,^^3^ GBS is a leading cause of life-threatening infections in newborns.^3^ Transmission of the bacteria from the mother to the baby can occur *in utero*, or during labor, and both routes are associated with early onset disease (EOD) within the first week of life. In addition, ascending infections during pregnancy can cause preterm delivery and stillbirth, and a conservative estimate is that GBS infections underlie 1–4% of all stillbirths.^3^^,^^4^^,^^5^ Current preventative measures involve intrapartum antibiotic prophylaxis (IAP); in the United States (US) it is offered to all pregnant women with GBS colonization confirmed by microbiological screening.^6^ The emergence of antibiotic resistance however imposes a threat to the IAP strategy,^7^ and intravenous delivery of antibiotics during labor is obviously not effective for preventing *in utero* infections or adverse pregnancy outcomes caused by ascending infections in the pregnant woman. IAP has also failed to reduce the incidence of neonatal late onset disease (LOD), presenting at 7–90 days of life.^8^ Novel prophylactic measures for prevention of adverse pregnancy outcomes and invasive neonatal disease caused by GBS therefore represents an unmet medical need, which may be addressed by a maternal vaccine that induces protective opsonophagocytic and placentally transferable antibodies.

The feasibility of a maternal GBS vaccine was demonstrated over 40 years ago,^9^ and since then several investigational and candidate capsular polysaccharide (CPS)-conjugate GBS vaccines have been assessed in clinical phase I and II studies.^10^^,^^11^^,^^12^^,^^13^^,^^14^^,^^15^^,^^16^ Analogous glycoconjugate vaccines have indeed proven efficacious for protection against *Streptococcus pneumoniae*, *Neisseria meningitidis*, and *Haemophilus influenzae*.^17^ In the case of maternal immunization, a protein vaccine might however prove more beneficial since proteins generally give rise to immunoglobulin G1 (IgG1) antibodies, which are more efficiently transferred across the placenta than the immunoglobulin G2 (IgG2) subclass that dominates responses against the CPS antigens.^18^^,^^19^

Essentially all GBS strains encode a surface protein belonging to the alpha-like protein (Alp) family.^20^ In total, six GBS Alp variants exist: Alpha C (αC), Rib, Alp1, Alp2, Alp3, and Alp4, of which Alp4 is extremely rare.^20^^,^^21^ They are encoded by mosaic gene variants at a single genomic locus, indicating that recombination of horizontally transferred gene fragments underlies the allelic diversity within the protein family.^22^ The Alps consist of a cell wall-anchored C-terminal domain, a 170 to 180 amino acid (aa) N-terminal domain, and an intervening repeat region with varying numbers of tandemly arranged repeat domains.^21^ Overall, there is 60%–70% sequence homology between the different Alp-Ns, and the sequences of Alp2-N and Alp3-N are identical.^21^ When present in the intact protein, the N-terminal domains of αC and Rib display immuno-sub-dominance relative to the immunodominant repeat regions, indicating that the bacteria have evolved an immune-evasion mechanism to avoid host responses against the N-terminal domains.^23^ This would in turn imply that the Alp-Ns are important for GBS virulence. Consistent with this idea, αC-N and Rib-N mediate GBS internalization into human cervical epithelial cells; in the case of αC-N, it is demonstrated to occur through interactions with the α_1_β_1_ integrin and glycosaminoglycan on the epithelial cells.^24^^,^^25^^,^^26^ In contrast to their poor immunogenicity in the presence of the repeat region, αC-N and Rib-N become highly immunogenic when engineered into a recombinant fusion protein lacking the remaining parts of the native proteins. Consequently, this fusion protein, termed GBS-NN, was shown to elicit protective immunity in adult mice^23^ and most recently also to confer protection against intranasal GBS challenge in neonatal mice born to immunized dams.^27^

We have recently reported that GBS-NN displays a good safety profile and is immunogenic in a randomized, placebo-controlled, double-blind phase I study involving 240 vaccinated adult healthy non-pregnant women.^28^ The antibody response induced by GBS-NN mediates opsonophagocytic killing and prevents GBS from invading human cervical epithelial cells.^29^ In addition to immunoglobulin G (IgG) and immunoglobulin A (IgA) against the vaccine constituents αC-N and Rib-N, the vaccine elicited responses against Alp1-N and Alp2/3-N not contained in the GBS-NN vaccine. These heterotypic responses were however more variable between subjects and correlated with homologous pre-existing immunity.^29^ To ensure robust responses against Alp1-N and Alp2/3-N also in immunologically naive subjects, we have now included a second fusion protein in the vaccine formulation, consisting of Alp1-N and Alp2/3-N (GBS-NN2). Here we report the results from a placebo-controlled double-blind phase 1 trial on the new two-component GBS-NN plus GBS-NN2 vaccine, which we have opted to call AlpN.

## Results

### Safety and tolerability

**Figure 1:**
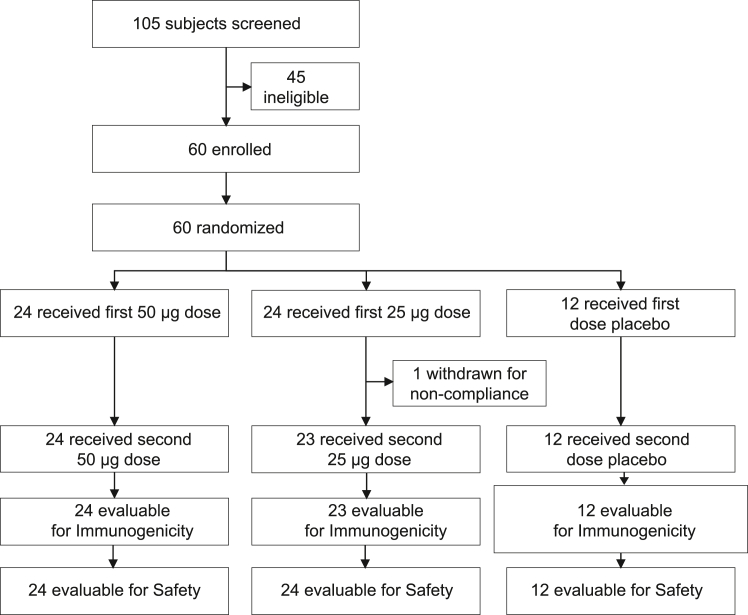
Trial profile of the clinical study Subject disposition of the clinical Alp-N vaccine study, outlining cohorts, dose regimens, and evaluation of immunogenicity and safety. See also Table S1.

The demographics of the study population are shown in Table 1. The disposition of subjects through the study is shown in Figure 1 and Table S1. The vaccine was well tolerated and had a good safety profile. There were no deaths and no serious adverse events (AEs) considered related to study medication following vaccination. There were also no early terminations or withdrawals due to vaccine safety or tolerability. Twelve subjects received placebo, and 48 subjects received active AlpN vaccine. Up to day 85 (twelve weeks after the first dose), a total of 89 treatment-emergent AEs (TEAEs) were reported by 40 (66.7%) subjects (Table 2). No severe AEs were reported; most AEs were graded as moderate and unrelated to the investigational medicinal product (IMP). Related TEAEs were reported by 7 (11.7%) subjects and included nausea, influenza-like illness, pyrexia, neck pain, dizziness, and headache, which, except for nausea, were considered systemic reactogenicity events (pre-defined, treatment-emergent events that occurred within two days of dosing). There were no treatment or dose-related trends, and a similar TEAE profile was observed for placebo and the cohorts receiving 25 μg or 50 μg AlpN. There were no clinically significant changes in serum biochemistry or hematological laboratory parameters. A low incidence of related systemic reactogenicity events (influenza-like illness, pyrexia, neck pain, dizziness, and headache) was reported. There was a low incidence of local reactogenicity (injection site reactions). Most subjects (approximately 83%) experienced no redness, bruising, induration, itching, or other local reactions, such as scratches, at the injection site.

**Table 1:** Summary of demography (ITT set)

Demography	Statistics	Placebo	AlpN (25 μg)	AlpN (50 μg)	AlpN overall	Overall
Subjects	N	12	24	24	48	60
Age (years)	Mean ± SD	27.3 ± 5.5	29.8 ± 5.4	30.8 ± 6.0	30.3 ± 5.7	29.7 ± 5.7
	Min/Median/Max	20/28.5/38	20/30.5/39	22/30.5/40	20/30.5/40	20/30/40
Weight (kg)	Mean ± SD	66.5 ± 10.2	65.6 ± 8.6	65.6 ± 9.1	65.6 ± 8.8	65.8 ± 9.0
Height (cm)	Mean ± SD	1.63 ± 0.1	1.65 ± 0.1	1.65 ± 0.1	1.65 ± 0.1	1.64 ± 0.1
BMI (kg/m^2^)	Mean ± SD	24.9 ± 2.5	24.1 ± 2.7	24.2 ± 2.8	24.2 ± 2.7	24.3 ± 2.7
Ethnic origin						
Caucasian	n (%)	12 (100)	23 (95.8)	24 (100)	47 (97.9)	59 (98.3)
Black	n (%)	0 (0)	1 (4.2)	0 (0)	1 (2.1)	1 (1.7)

**Table 2:** TEAEs in each treatment group by system organ class and preferred term up to day 85 (safety set)

System organ class	Number (%) of subjects				
Preferred term	Placebo (N = 12)	AlpN (25 μg) (N = 24)	AlpN (50 μg) (N = 24)	AlpN Overall (N = 48)	Overall (N = 60)
**Cardiac disorders**					

Palpitations	0 (0.0)	1 (4.2)	0 (0.0)	1 (2.1)	1 (1.7)

**Gastrointestinal disorders**					

Abdominal distension	0 (0.0)	0 (0.0)	1 (4.2)	1 (2.1)	1 (1.7)
Dyspepsia	0 (0.0)	1 (4.2)	0 (0.0)	1 (2.1)	1 (1.7)
Nausea	0 (0.0)	1 (4.2)	2 (8.3)	3 (6.3)	3 (5.0)
Vomiting	0 (0.0)	0 (0.0)	1 (4.2)	1 (2.1)	1 (1.7)

**General disorders and administration site conditions**					

Influenza-like illness	0 (0.0)	2 (8.3)	1 (4.2)	3 (6.3)	3 (5.0)
Pain	1 (8.3)	0 (0.0)	0 (0.0)	0 (0.0)	1 (1.7)
Pyrexia	0 (0.0)	1 (4.2)	0 (0.0)	1 (2.1)	1 (1.7)

**Immune system disorders**					

Allergy to animal	0 (0.0)	1 (4.2)	0 (0.0)	1 (2.1)	1 (1.7)

**Infections and infestations**					

Gastroenteritis viral	1 (8.3)	1 (4.2)	0 (0.0)	1 (2.1)	2 (3.3)
Influenza	0 (0.0)	2 (8.3)	0 (0.0)	2 (4.2)	2 (3.3)
Lower respiratory tract infection	0 (0.0)	1 (4.2)	0 (0.0)	1 (2.1)	1 (1.7)
Nasopharyngitis	2 (16.7)	3 (12.5)	2 (8.3)	5 (10.4)	7 (11.7)
Pharyngitis	0 (0.0)	1 (4.2)	0 (0.0)	1 (2.1)	1 (1.7)
Tonsillitis	0 (0.0)	0 (0.0)	1 (4.2)	1 (2.1)	1 (1.7)
Upper respiratory tract infection	0 (0.0)	0 (0.0)	1 (4.2)	1 (2.1)	1 (1.7)
Urinary tract infection	0 (0.0)	1 (4.2)	1 (4.2)	2 (4.2)	2 (3.3)
Viral infection	1 (8.3)	0 (0.0)	0 (0.0)	0 (0.0)	1 (1.7)
Vulvovaginal candidiasis	0 (0.0)	2 (8.3)	0 (0.0)	2 (4.2)	2 (3.3)

**Injury, poisoning, and procedural complications**					

Ligament sprain	0 (0.0)	0 (0.0)	1 (4.2)	1 (2.1)	1 (1.7)
Post-traumatic neck syndrome	0 (0.0)	1 (4.2)	0 (0.0)	1 (2.1)	1 (1.7)

**Musculoskeletal and connective tissue disorders**					

Arthralgia	0 (0.0)	0 (0.0)	1 (4.2)	1 (2.1)	1 (1.7)
Back pain	0 (0.0)	1 (4.2)	0 (0.0)	1 (2.1)	1 (1.7)
Musculoskeletal chest pain	0 (0.0)	1 (4.2)	0 (0.0)	1 (2.1)	1 (1.7)
Neck pain	1 (8.3)	0 (0.0)	0 (0.0)	0 (0.0)	1 (1.7)
Pain in extremity	0 (0.0)	0 (0.0)	1 (4.2)	1 (2.1)	1 (1.7)

**Nervous system disorders**					

Carpal tunnel syndrome	0 (0.0)	1 (4.2)	0 (0.0)	1 (2.1)	1 (1.7)
Dizziness	1 (8.3)	0 (0.0)	0 (0.0)	0 (0.0)	1 (1.7)
Headache	5 (41.7)	9 (37.5)	5 (20.8)	14 (29.2)	19 (31.7)
Lethargy	0 (0.0)	1 (4.2)	1 (4.2)	2 (4.2)	2 (3.3)
Migraine	0 (0.0)	0 (0.0)	1 (4.2)	1 (2.1)	1 (1.7)

**Psychiatric disorders**					

Anxiety	1 (8.3)	0 (0.0)	0 (0.0)	0 (0.0)	1 (1.7)
Sleep disorder	0 (0.0)	0 (0.0)	1 (4.2)	1 (2.1)	1 (1.7)

**Reproductive system and breast disorders**					

Dysmenorrhea	0 (0.0)	3 (12.5)	1 (4.2)	4 (8.3)	4 (6.7)
Hypomenorrhea	1 (8.3)	0 (0.0)	0 (0.0)	0 (0.0)	1 (1.7)

**Respiratory, thoracic and mediastinal disorders**					

Cough	0 (0.0)	1 (4.2)	0 (0.0)	1 (2.1)	1 (1.7)
Oropharyngeal pain	1 (8.3)	0 (0.0)	2 (8.3)	2 (4.2)	3 (5.0)

A subject was counted only once per system organ class and preferred term within each treatment category.% were calculated from the number of subjects in the safety set at each specific treatment group.

Pain was the commonest reported injection site reaction, with the majority of participants providing at least one report of pain. For both placebo and the two-dose levels of the vaccine, transient pain (mainly mild to moderate) tended to be experienced, more frequently, at the injection site the day after dosing (day 2 and 30, respectively), compared to the day of dosing. Approximately 28% of subjects receiving the vaccine experienced injection site pain immediately after administration of the second dose of AlpN (day 29); only one subject (4.2%) reported pain immediately after the first injection. Following the second dose, reports of pain were less frequent overall on the day after dosing than on the day after subjects received the first dose. Approximately 48% of subjects reported pain on day 2 (the day after the first dose) compared to approximately 36% reporting pain on day 30 (the day after the second dose).

In summary, most AEs were related to injection site events and the majority of all AEs were of moderate intensity.

### Immunogenicity

#### Vaccination with AlpN induces persistent IgG responses against both GBS-NN and GBS-NN2

**Figure 2:**
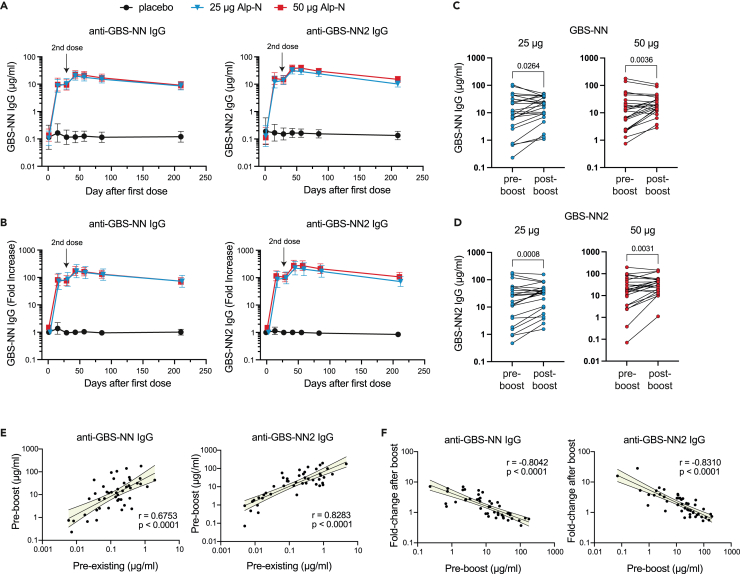
IgG responses against the vaccine constituent GBS-NN and GBS-NN2 Subjects were immunized with two doses of 25 or 50 μg of Alp-N together with the adjuvant AlOH as indicated in the legend (n = 23 per dose level). The second dose was administered 28 days after primary vaccination. Subjects in the placebo group received two doses of adjuvant only (n = 12). (A and B) GBS-NN- and GBS-NN2-specific IgG GMC (A) and fold change relative to pre-vaccination level (B) for indicated time points. Error bars show 95% CI. (C and D) Paired t-test analyses of individual pre-boost (day 29) and post-boost (day 85) levels of IgG against GBS-NN (C) and GBS-NN2 (D) for indicated dose levels. (E and F) Pearson correlation plots with 95% CI bands, showing the associations between concentration of pre-existing and pre-boost (day 29) IgG concentrations (E) and between pre-boost IgG concentrations (day 29) and boost effect of a second dose (fold change day 85 relative to day 29) (F). Pearson correlation analyses performed on all subjects combined from the 25 μg and 50 μg cohorts (n = 46). All statistical analyses performed on logarithmically transformed data. See also Figure S1.

Vaccination with the AlpN vaccine resulted in significantly increased geometric mean concentrations (GMC) of IgG against both GBS-NN and GBS-NN2, whereas no such increase was observed for the placebo group (Figure 2A). Already two weeks after the first dose (day 15), levels of GBS-NN-specific IgG were increased 70- and 80-fold relative to baseline for the 25 and 50 μg cohort, respectively (geometric mean fold increases; Figure 2B). At the same time, GBS-NN2-specific IgG levels increased 86-fold for the lower and 116-fold for the higher dose level (Figure 2B). Specific IgG levels then remained unchanged for two additional weeks prior to boosting (no significant differences detected within the cohorts between day 15 and day 29; Figure S1). Following administration of the second dose four weeks after the primary course, both GBS-NN and GBS-NN2-specific responses were further boosted at least 2-fold, with specific IgG GMCs peaking 2–4 weeks after boosting (day 43 and 57 relative to the primary dose, respectively) (Figures 2A and 2B). Significantly elevated levels of IgG against both vaccine components (>70-fold relative to baseline) were thereafter maintained throughout the 210 days duration of the study (Figures 2A and 2B). There was no significant difference in IgG GMC or fold change between the 25 and 50 μg cohorts at any time point.

The IgG concentrations twelve weeks after the first dose (day 85; 8 weeks after boost) constituted the primary immunological endpoint, corresponding to the expected time of birth if the vaccine had been given at the start of the third trimester of pregnancy. IgG GMCs 8 weeks after the boost were slightly reduced compared to the peak response 2–4 weeks post-secondary immunization (Figure 2A). Even if specific IgG levels accordingly had started to drop somewhat at this time point in some of the subjects, paired analyses of individual subject IgG concentrations before (day 29) and eight weeks after (day 85) administration of the second dose confirmed a significant boost effect for both fusion proteins also at this later time point (Figures 2C and 2D). Given that specific IgG responses peaked already 2–4 weeks after the second dose, even stronger boost effects were observed when similar analyses were performed for these earlier time points (Figure S1).

Altogether these results demonstrate that vaccination with AlpN elicits robust and persistent IgG responses against both fusion proteins contained in the vaccine, that vaccination with 25 or 50 μg results in similar IgG GMCs, and, finally, that all responses are significantly boosted by a second dose administered four weeks after primary immunization.

#### Boost effect of the second dose is primarily driven by an added beneficial effect in subjects with low pre-existing immunity

A more detailed examination of the results, as presented in Figures 2C and 2D and in Figure S1, indicated a stratification of the cohorts into subjects reaching a saturated response already after the first dose versus vaccinees with a relatively poor primary response but with a clear beneficial effect of the second dose. To better understand the importance of a second dose in relation to pre-existing immunity and the magnitude of the primary vaccine response, we performed Pearson correlation analyses after pooling results from the 25 and 50 μg cohorts (n = 48). For both GBS-NN and GBS-NN2, the concentration of IgG induced by primary vaccination (pre-boost response; day 29) correlated with the levels of pre-existing IgG against the respective protein (Figure 2E). Furthermore, for both fusion proteins, there was a strong inverse correlation between the pre-boost IgG concentrations (day 29) and the ability of the second dose to boost the response, as measured eight weeks after secondary immunization (day 85; the primary immunological endpoint of the study) (Figure 2F). In conclusion, the added beneficial effect of a second dose is driven primarily by improved responses in subjects responding relatively weakly to the first dose, which in turn is associated with low pre-existing immunity.

#### The AlpN vaccine elicits IgG1 and IgA responses to all individual Alp-N domains

**Figure 3:**
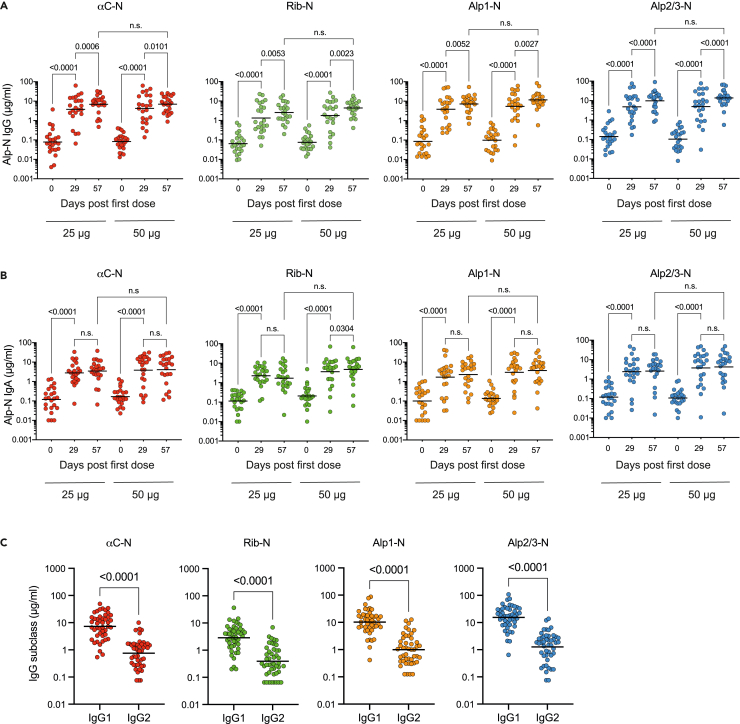
IgG and IgA responses against individual Alp-N domains (A and B) Serum concentrations of IgG (A) and IgA (B) against indicated Alp-N domains were determined by ELISA separately for the cohorts receiving 25 μg and 50 μg of the vaccine as indicated (n = 23 for each cohort). Results show individual subject concentrations and GMCs before vaccination (day 0), after the first dose (day 29), and four weeks after the second dose (day 57). Paired t-tests were used for intra-cohort comparisons. Unpaired t-tests were used for inter-cohort comparisons. (C) Serum concentrations of IgG1 and IgG2 against indicated Alp-N domains four weeks after the second dose (day 57). Paired t-tests performed on all subjects combined from the 25 μg and 50 μg cohorts, showing individual subject concentrations and GMCs (n = 46). All statistical analyses performed on logarithmically transformed data.

As each of the two vaccine antigens is a fusion protein comprising N-terminal domains of two Alp family members, we next determined the antibody levels against all four individual Alp-Ns present in the vaccine. Consistent with our phase I study on the single component GBS-NN vaccine,^28^ for all domains mostly low but variable levels of IgG and IgA were present in all pre-vaccination sera (Figures 3A and 3B; Table 3). Primary vaccination induced significant IgG and IgA responses against each of the domains (Figures 3A and 3B). In line with the IgG response against GBS-NN and GBS-NN2, the second dose resulted in significantly increased GMCs of IgG against all individual Alp-Ns, as assessed four weeks after the second dose (Figures 3A and Table 3). The GMCs of specific IgG after two doses of 50 μg Alp-N reached 7.8 μg/mL for αC-N, 4.4 μg/mL for Rib-N, 11.7 μg/mL for Alp1-N, and 13.8 μg/mL for Alp2/3-N. There were no significant differences in the GMC of IgG against any of the four individual Alp-Ns when comparing the 25 μg and 50 μg dose levels (Figure 3A), again in line with the IgG response against GBS-NN and GBS-NN2. As previously reported for the GBS-NN component alone,^28^^,^^29^ the IgG response was strongly dominated by IgG1. For all Alp-Ns, the concentration of IgG1 was at least 10-fold higher than the homologous IgG2 levels (Figure 3C). In contrast to IgG, the robust IgA responses observed after primary immunization were not further increased following administration of the second dose (Figure 3B), reiterating the lack of boost effect previously observed for IgA responses against the single component GBS-NN vaccine.^28^ Collectively, these results demonstrate that AlpN induces robust primary IgG1 and IgA responses to all four individual Alp-Ns contained in the vaccine, with a clear beneficial effect of a second dose on the IgG but not on the IgA responses.

**Table 3:** Alp-N-specific IgG and IgA serum concentration pre-vaccination (day 1), after the first dose (day 29), and after the second dose (day57) of AlpN

Cohort	Ab Response	αC-N			Rib-N			Alp1-N			Alp2/3-N		
		Day 1	Day 29	Day 57	Day 1	Day 29	Day 57	Day 1	Day 29	Day 57	Day 1	Day 29	Day 57
**25 μg Dose**	**IgG (μg/mL)**	**0.08**	**3.77**	**6.94**	**0.06**	**1.34**	**2.57**	**0.09**	**3.82**	**7.16**	**0.14**	**4.80**	**9.73**

	95% CI	(0.04-0.15)	(1.79-7.92)	(4.22-11.4)	(0.04-0.11)	(0.62-2.87)	(1.57-4.21)	(0.04-0.16)	(1.84-7.94)	(4.62-11.1)	(0.08-0.25)	(2.36-9.77)	(5.85-16.2)
	Fold change^a^	–	48	89	–	21	40	–	44	83	–	34	68
	95% CI	–	(26–90)	(52–152)	–	(10–43)	(22–72)	–	(25–78)	(52–131)	–	(20–56)	(46–101)

	**IgA (μg/mL)**	**0.12**	**2.78**	**3.47**	**0.11**	**2.27**	**1.71**	**0.10**	**1.69**	**2.30**	**0.12**	**2.44**	**2.61**

	95% CI	(0.06-0.22)	(1.59-4.89)	1.92-6.29)	(0.07-0.18)	(1.35-3.80)	(0.90-3.23)	(0.05-0.20)	(0.71-4.02)	(1.17-4.53)	(0.06-0.22)	(1.07-5.54)	(1.24-5.46)
	Fold change^a^	–	23	29	–	20	15	–	17	23	–	21	22
	95% CI	–	(11–51)	(16–54)	–	(10–39)	(8–29)	–	(8–37)	(12–45)	–	(10–43)	(12–42)

**50 μg Dose**	**IgG (μg/mL)**	**0.08**	**4.23**	**7.77**	**0.08**	**1.80**	**4.40**	**0.10**	**5.42**	**11.7**	**0.10**	**4.92**	**13.8**

	95% CI	(0.05-0.12)	(2.03-8.82)	(5.07-11.9)	(0.05-0.11)	(0.84-3.90)	(2.95-6.55)	(0.06-0.17)	(2.55-11.5)	(7.65-17.8)	(0.06-0.18)	(2.16-11.2)	(8.75-21.8)
	Fold change^a^	–	51	94	–	24	58	–	50	114	–	47	132
	95% CI	–	(31–85)	(69–128)	–	(12–49)	(37–91)	–	(32–77)	(81–160)	–	(30–76)	(101–174)

	**IgA (μg/mL)**	**0.17**	**3.85**	**4.06**	**0.21**	**3.68**	**4.79**	**0.14**	**3.00**	**3.69**	**0.11**	**3.77**	**4.25**

	95% CI	(0.11-0.26)	(1.72-8.62)	(1.93-8.56)	(0.12-0.35)	(1.79-7.57)	(2.50-9.16)	(0.09-0.20)	(1.37-6.60)	(1.88-7.24)	(0.07-0.17)	(1.55-9.19)	(1.97-9.17)
	Fold change^a^	–	23	24	–	18	23	–	22	27	–	35	39
	95% CI	–	(13–40)	(15–38)	–	(9–35)	(12–43)	–	(12–39)	16-45)	–	(19–63)	(24–66)

a Fold change relative to pre-vaccination (day 1) serum concentration.

#### A second vaccine dose lifts more subjects above pre-defined Alp-N-specific IgG thresholds

As protective thresholds for IgG against each Alp-N domain remain to be firmly established, we next determined the percentage of subjects reaching the arbitrary set thresholds of 0.5, 1.0, and 2.0 μg/mL IgG against each of the domains. For the cohort receiving two 50 μg doses of the vaccine, 100% of subjects reached ≥0.5 μg/mL of IgG against αC-N, Alp1, and Alp2/3-N and 96% of subjects reached ≥0.5 μg/mL of IgG against Rib-N. For all individual Alp-Ns, 96% of the subjects reached ≥1 μg/mL of IgG and most subjects achieved specific IgG concentrations also above the 2 μg/mL threshold (Table 4). The proportions of subjects achieving IgG concentrations above these pre-defined thresholds were higher overall in the group that received 50 μg than in the group receiving 25 μg of the vaccine (Table 4). These differences were however not statistically significant when analyzed by Fisher exact test. Finally, the proportions of subjects reaching the threshold of 0.5 μg/mL IgG after the first 50 μg dose was 78% for Rib-N and around 90% for the remaining Alp-Ns (Table S2). Given that close to 100% of the subjects reached the same thresholds after the second dose, approximately 10–20% of the subjects benefited from the booster dose in terms of reaching the 0.5 μg/mL thresholds. Similarly, 20–30% of the subjects in this cohort benefited from the second dose in terms of reaching the 1.0 μg/mL thresholds (Table S2).

**Table 4:** Percent subjects in the 25 μg and 50 μg cohorts reaching pre-defined thresholds of Alp-N-specific IgG after the second dose (Day 57)

Specificity	Alp-N specific IgG thresholds per vaccine dose level					
	>0.5 μg/mL		>1 μg/mL		>2 μg/mL	
	25 μg	50 μg	25 μg	50 μg	25 μg	50 μg
αC-N	96%	100%	91%	96%	83%	91%
Rib-N	91%	96%	83%	96%	52%	83%
Alp1-N	100%	100%	96%	96%	87%	96%
Alp2/3-N	100%	100%	91%	96%	91%	96%

See also Table S2.

#### Enhanced opsonophagocytic killing of all vaccine-homotypic Alp-N serotypes following vaccination with the Alp-N vaccine

The ability of the antibody response induced by the Alp-N vaccine to mediate opsonophagocytic killing was assessed in the opsonophagocytic killing assay (OPkA) adapted from Burton and Nahm, using baby rabbit complement and differentiated human HL-60 cells as fixed sources of complement and neutrophil-like cells, respectively.^30^ We restricted our analysis to the 50-μg dose cohort, and GBS strains were selected to include all Alp-N serotypes covered by the vaccine: A909 (αC; CPS Ia), BM110 (Rib; CPS III), NCTC12906 (Alp1; CPS Ia), and NEM316 (Alp2; CPS III). Given the identical sequence of Alp2-N and Alp3-N,^21^ we assessed responses against Alp2 and Alp3 collectively by measuring killing of the Alp2-expressing strain NEM316.

**Figure 4:**
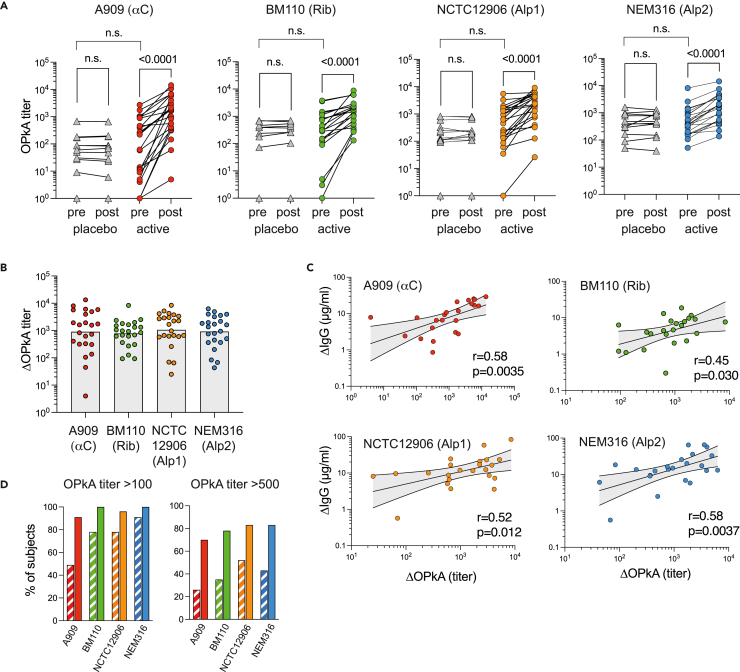
OPk responses against all four Alp-N serotypes covered by the Alp-N vaccine Pre- and day 57 post-vaccination sera from all subjects of the 50-μg dose cohort (n = 23) and placebo (n = 12) were assessed for the ability to mediate OPk of the indicated GBS strains. OPkA titer is defined as the reciprocal serum dilution required to mediate 50% bacterial killing relative to killing in the absence of human serum. (A) Total OPk titers achieved against indicated GBS strains. Results show paired t-tests for individual pre- and post-vaccination sera. Unpaired t-tests were used for comparison of placebo versus active pre-vaccination sera. (B) ΔOPkA titers (derived by subtracting the pre-vaccination titer from the paired post-vaccination titer) against indicated strains. Results show individual ΔOPkA titers and ΔOPkA GMTs. (C) Pearson correlation plots with 95% CI bands, showing ΔOPkA titers versus the Alp-N homologous IgG response induced by the vaccine (ΔIgG) for the indicated GBS target strains. (D) Percent of subjects reaching the OPkA titer thresholds >100 and >500 against indicated GBS strains before (striped bars) and after (solid bars) two 50 μg doses of AlpN. All statistical analyses performed on logarithmically transformed data. See also Table S3.

Most subjects had measurable but variable OPkA titers against all strains already before vaccination (Figure 4A and Table 5), probably reflecting pre-existing IgM and IgG antibodies specific for a wide range of target molecules on the bacterial cell surface (including multiple protein antigens as well as CPS). Titers against all four strains were increased in all subjects following vaccination, giving rise to significantly increased OPkA geometric mean titers (GMTs) four weeks after the second dose, as compared to corresponding pre-vaccination GMTs (Figures 4A and Table 5). No such increase was observed for the placebo group (Figure 4A). For subjects receiving the vaccine, the titers were approximately 16-, 6-, 5-, and 3-times higher against A909 (αC), BM110 (Rib), NCTC12906 (Alp1), and NEM316 (Alp2), respectively, following vaccination compared to pre-vaccination (geometric mean fold increases; Table 5). Importantly, the relatively wide range in fold-increase observed against the different strains was not due to variations in how efficiently the vaccine enhanced the neutrophil-mediated killing of the different GBS Alp serotypes but was instead related to variations in the pre-existing OPkA activity against the four target strains. This was evident from the ΔOPkA titers, the vaccine-induced increase in OPkA titers calculated by subtracting individual pre-from post-vaccination titers. As shown in Figure 4B, the ΔOPkA GMTs against the four GBS target strains were remarkably similar; ranging from 785 to 1060 for the four separate target strains (Table 5). Furthermore, individual subject ΔOPkA titers displayed moderate correlations with the homologous Alp-N IgG concentrations induced by the vaccine (ΔIgG) (Figure 4C), indicating that the opsonophagocytic killing activity conferred by the vaccine with some confidence can be estimated from the Alp-N-specific IgG serum concentrations measured by ELISA (specific killing activity per μg/IgG; ΔOPkA/ΔIgG values in Table 5). Finally, vaccination resulted in that essentially all subjects reached an arbitrarily set OPkA titer threshold of >100 against all the four Alp serotypes, and the proportion of subjects reaching the titer threshold of >500 was roughly doubled for all the strains following vaccination (Figure 4D).

**Table 5:** OPkA titers before (day 1) and after (day 57) vaccination with two doses of 50 μg AlpN (n = 23) assessed against indicated GBS strains (Alp serotypes)

GBS strain	OPkA titer Day 1	OPkA titer Day 57	OPkA Fold change^a^	ΔOPkA titer (units/mL)^b^	ΔIgG (μg/mL)^c^	ΔOPkA/ΔIgG (units/μg)
(Alp-N/CPS)	GMT^d^ (95% CI)	GMT^d^ (95% CI)	GM^e^ (95% CI)	GMT^d^ (95% CI)	GMC^f^ (95% CI)	GM^e^ (95% CI)
A909 (αC/Ia)	72 (23, 220)	1145 (513, 2552)	15.9 (7.0, 36.3)	902 (394, 2064)	7.7 (5.0, 11.8)	122 (65, 223)
BM110 (Rib/III)	219 (89, 540)	1240 (780, 1970)	5.6 (3.1, 10.4)	785 (488, 1262)	4.2 (2.8, 6.4)	184 (116, 292)
NCTC12906 (Alp1/Ia)	378 (168, 846)	1807 (977, 3344)	4.8 (3.0, 7.8)	1060 (549, 2046)	11.1 (7.2, 17.2)	95 (54, 169)
NEM316 (Alp2/III)	450 (276, 733)	1503 (899, 2512)	3.3 (2.6, 4.2)	918 (499, 1689)	13.7 (8.7, 21.6)	67 (40, 111)

See also Table S3.

a Fold change in titer day 57 relative day 1.

b ΔOPkA titer = OPkA titer (day 57-day 1).

c ΔIgG = Alp-N homologous IgG serum concentration (day 57-day1).

d GMT; Geometric mean titer.

e GM; Geometric mean.

f GMC; Geometric mean concentration.

Next, we assessed if antibodies induced by the vaccine could mediate opsonophagocytic killing of GBS strains distinct from the four reference strains used in our standard OPkA. To this end, we opted to use a panel of five GBS strains selected to cover the most clinically relevant CPS types in a standardized OPkA assay that is currently under development by the GASTON consortium^31^: National Collection of type Cultures (NCTC) strains 14092 (αC; CPS Ib), 14091 (Rib; CPS III), 14093 (Rib; CPS II), 14094 (Alp1; CPS Ia), and 14095 (Alp2; CPS V). Of note, the αC-, the Alp2-, and one of the Rib-expressing strains in this panel possess different CPS types than the Alp homologous strains assessed in the experiments described above. Using two pooled sera, prepared from equal volumes of either pre- or post-vaccination sera from seven high-titer vaccinated subjects, the ΔOPkA titer achieved against each of these GASTON GBS strains was similar to the ΔOPkA titer achieved against the Alp homologous reference strain assayed in parallel against the same pooled sera (Table S3).

Together these results show that the AlpN vaccine elicits an opsonophagocytic serum response that is proportional to the Alp-N homologous IgG response and which is equally efficacious against GBS strains of all Alp-N serotypes covered by the vaccine.

## Discussion

The current results extend those reported previously on the study involving 240 women vaccinated with various doses of the GBS-NN component alone or placebo.^28^^,^^29^ The safety profile of the new two-component AlpN vaccine at total protein doses of 50 μg (2 × 25 μg) or 100 μg (2 × 50 μg) is comparable to other aluminum-adjuvanted vaccines and in line with our previous study on the GBS-NN component alone, at doses up to 250 μg.^28^ Our study shows that AlpN is highly immunogenic at doses of 25 or 50 μg and that vaccination gives rise to robust and persistent antibody responses against all Alp-Ns contained in the vaccine, with trends toward enhanced responses following administration of the 50-μg compared to the 25-μg dose level.

Although we observed significant antibody responses against Alp1-N and Alp2/3-N in our previous study of the single-component GBS-NN vaccine (containing only the αC-N and Rib-N fusion protein), the magnitude of these responses varied considerably between individual study subjects.^29^ Strikingly, even after administration of the second GBS-NN dose, the Alp1-N- and Alp2/3-N-specific IgG concentrations correlated strongly with levels of homologous pre-existing antibodies. This means that individuals with low level of naturally acquired antibodies against Alp1-N or Alp2/3-N, and hence most at risk for infections caused by GBS strains expressing any of these Alp alleles, would most likely not achieve sufficiently high levels of antibodies against Alp1-N and Alp2/3-N following GBS-NN vaccination. Accordingly, we have now included a second fusion protein consisting of Alp1-N and Alp2/3-N in the new vaccine formulation. Our results demonstrate that this second-generation two-component vaccine elicits a robust IgG response against all four Alp-Ns. For example, in the current study, the GMC of IgG against Alp2/3-N reached 13.8 μg/mL, which should be compared to the 2.42 μg/mL achieved in our former phase I study on GBS-NN.^29^ The elevated response observed against Alp1-N and Alp2/3-N after adding GBS-NN2 to the vaccine formulation did not occur at the expense of responses against αC-N or Rib-N. Instead, GMCs of IgG against the latter domains were also increased relative to the corresponding GMC values previously achieved after two doses with the GBS-NN vaccine (7.8 μg/mL versus 5.5 μg/mL for αC-N and 4.4 μg/mL versus 2.2 μg/mL for Rib-N).^29^ The robust IgG responses observed after two doses of the new AlpN vaccine formulation therefore predict a broad coverage against all invasive isolates. Indeed, at least one of the five Alps covered by the current vaccine formulation (αC, Rib, Alp1, Alp2, and Alp3) could be detected in 99.3% of 6,340 invasive GBS isolates collected in the US during 2015–2017.^20^

Our previous study on GBS-NN included a cohort receiving only one dose of the vaccine.^28^ This allowed for a direct comparison between one- and two-dose regimens, demonstrating substantially higher IgG GMCs for the cohorts receiving two doses for the whole one-year duration of the study. Based on those results, we did not include a single-dose cohort in the current study. To evaluate the effects of a second dose, we have here instead performed paired analyses of IgG concentrations, measured in the same subjects before and after receiving the second dose. Although this approach suffers from the fact that responses after one versus two doses cannot be compared at the same time point post-primary immunization (and hence will not reflect the decay that would occur at later time points also for a cohort receiving only one dose), our results still demonstrate significantly higher IgG responses four weeks after the second dose as compared to four weeks after the first dose. Specific IgG levels then started to decline, resulting in that some subjects, who achieved very high and seemingly saturated titers already after primary immunization, presented concentrations even below post-primary levels when assessed 8 weeks after the second dose (corresponding to the primary immunological study endpoint). However, at the cohort level, titers were still significantly higher 8 weeks after, as compared to before, administration of the second dose. These analyses therefore corroborate results from our former study, demonstrating that significantly higher IgG GMCs are achieved with a two-dose regimen.

A critical measure of the likely success of the vaccine in preventing disease in newborn babies will be its ability to broadly induce concentrations of antibodies against the individual Alp-N serotypes above a protective threshold. Lifting specific serum IgG titers above such protective threshold in as many vaccinees as possible represents a more important outcome of a vaccination program than to induce high GMCs for the entire cohort. GBS isolates expressing either Rib or Alp1 accounts for 64% of EOD cases and 85% of LOD cases in the US.^20^ Preliminary 90% protective correlate of protection (CoP) thresholds of 0.43 μg/mL for Rib-N neonatal IgG and 0.11 μg/mL for Alp1-N neonatal IgG have recently been suggested (Dangor et al. 2nd International Symposium on *Streptococcus agalactiae* Disease [ISSAD, Nov 3-5, 2021]). No such thresholds have yet been assessed for αC-N and Alp2/3-N, due to a lower prevalence of such clinical isolates.^20^ Following vaccination with a single dose of 50 μg AlpN, 78% of subjects reached >0.5 μg/mL Rib-N-specific IgG, corresponding roughly to the 90% CoP mentioned above. After the second 50 μg dose, 96% of subjects reached >1 μg/mL, more than a 2-fold higher concentration relative to the proposed 90% CoP for neonatal Rib-N IgG. Furthermore, for all individual Alp-Ns, our results show that 20–30% of the subjects receiving the 50 μg dose level benefit from a second dose in terms of reaching the arbitrary >1 μg/mL threshold and that this effect primarily is driven by a pronounced boosting in subjects with low pre-existing immunity and hence most at risk. Whether or not the vaccine will ultimately be given as a single- or two-dose regimen depends on the level of validated CoPs currently being developed. If current preliminary thresholds are maintained, combined with the fact that the AlpN vaccine results in primarily IgG1 antibodies that may accumulate in neonatal relative maternal blood (see below), a single-dose vaccine may well be developed. However, the current results demonstrate that a booster dose will most likely under all circumstances help the subjects with the lowest pre-vaccination antibody levels to reach the protective threshold.

Antibody-mediated opsonization, coupled with phagocytic killing by neutrophils, represents a key effector mechanism of the immune system for preventing bodily dissemination of Gram-positive bacteria such as *Streptococcus pneumoniae* and GBS. Results from both this study and our previous clinical trial on the single-component prototype GBS-NN vaccine reveal that a surprisingly high proportion of the vaccinees exhibit relatively high OPkA serum titers against GBS already before vaccination. While this appears to stand in contrast to the relatively low levels of pre-existing IgG against the Alp-Ns, the OPkA does not discriminate between antibodies specific for different GBS antigens but instead reports bacterial killing mediated by all opsonic GBS-binding antibodies present in the sera. The pre-existing OPkA titers are therefore likely to reflect antibodies directed against multiple surface proteins, including the intact Alps (also containing the repeat regions) and the GBS pilus protein,^32^^,^^33^ as well as against the CPS.^16^^,^^32^ In the context of pregnancy, it remains unclear to what extent these naturally acquired and opsonophagocytic antibodies will be available for neonatal protection as a large proportion may consist of IgM, which is not transferred across the placenta,^18^ or of IgG subclasses exhibiting poor transfer rate, including IgG2.^34^ In addition, even though we show relatively high pre-existing OPkA GMTs, some subjects still exhibited very low or non-detectable OPkA titers prior to vaccination. Inducing a placentally transferable opsonophagocytic killing response in these subjects is probably the most important goal for a maternal vaccination program.

AlpN vaccination resulted in increased OPkA serum titers in every single subject of the cohort, resulting in that essentially all subjects reached titers >100 against all Alp-N serotypes. At the cohort level, the magnitude of the opsonophagocytic killing response was similar for all GBS Alp-N serotypes tested. As all Alps, except for the extremely rare Alp4 allele, are represented by the four Alp-Ns contained in vaccine, the results indicate that the antibody response may be equally efficacious against all clinically relevant Alp serotypes. Furthermore, the increase in OPkA titers following AlpN vaccination (i.e., ΔOPkA titers) correlated with the homologous Alp-N-specific IgG response induced by the vaccine, and even stronger correlations were observed in our previous study on GBS-NN, probably reflecting the larger cohorts assessed in that study.^29^ The association between IgG responses and increased OPkA titers indicates that the increased OPk activity in post-vaccination sera is primarily due to IgG rather than IgM. Consistent with this, we have recently shown that the single-component GBS-NN vaccine induces very little Alp-N-specific IgM and that the increased OPk activity observed following GBS-NN vaccination correlates only poorly or not at all with IgM against αC-N or Rib-N, respectively.^29^ Further along these lines, our current results show that the IgG response against each individual Alp-N domain is strongly dominated by IgG1, with only minor contribution of IgG2. Given that IgG1 is transferred more efficiently than IgG2 across the placenta,^34^ our results indicate that the serum concentrations of opsonophagocytic IgG induced in the non-pregnant women enrolled to the current study might reflect levels of functionally active IgG ultimately available for protection in neonates following maternal vaccination. Consistent with this idea, naturally acquired IgG against αC-N and Rib-N, which also consists of mostly IgG1, is present at higher concentrations in cord relative to postpartum maternal blood,^29^ and maternal immunization with protein-based vaccines leads to an accumulation of specific IgG in the neonatal relative to maternal blood.^35^^,^^36^

Our results further suggest that the CPS serotypes expressed by the strains do not have an impact on how efficiently a given Alp serotype is targeted by the vaccine response. This is illustrated by the finding that vaccination enhanced opsonophagocytic killing to the same extent when comparing αC-, Rib-, or Alp2-expressing strains co-expressing CPS of different serotypes. These results extend our previous findings that antibodies induced by the GBS-NN vaccine mediate efficient killing of αC-expressing clinical isolates collected from cases of EOD, irrespective of their capsule type.^29^

In summary, this study shows that the AlpN vaccine is safe and elicits a persistent and opsonophagocytic antibody response that efficiently targets all Alps covered by the vaccine. The opsonophagocytic response induced by the vaccine correlates with the Alp-N homologous IgG response, and placentally transferable IgG1 is the most dominant IgG subclass induced. The higher dose level of 50 μg AlpN shows a trend toward higher percentages of subjects reaching arbitrarily set serum IgG concentration thresholds. Further development of two AlOH-adjuvanted 50 μg doses of the vaccine is supported.

### Limitations of the study

A limitation of this study is that the clinical trial did not include a cohort receiving only one dose of the AlpN vaccine. To determine if a second dose is required to reach adequate protection, cohorts receiving one versus two doses of AlpN need to be directly compared in respect of both specific IgG responses and a functional surrogate of protection, i.e. OPkA. Given the pronounced pre-existing OPkA activity in blood of adult healthy women, which may be transferred poorly to the fetus, measurements of OPkA responses in neonatal blood is more likely to reflect neonatal protection achieved after maternal immunization. A phase II study involving cohorts of pregnant women receiving one or two doses of AlpN, which will include OPkA analyses on neonatal blood samples, has therefore been initiated.

## STAR★Methods

### Key resources table

**Table:** 

REAGENT or RESOUR	SOURCE	IDENTIFIER
**Antibodies**		

Subcuvia 160mng/mL	Baxalta, Sweden	Lot: VNGN018A
Goat anti-human IgG (γ-chain) F(ab’)2-HRP	SouthernBiotech	Cat#2042-05; RRID:AB_2795660
Goat anti-human IgA (α-chain)-HRP	SouthernBiotech	Cat#2050-05; RRID:AB_2687526
Mouse anti-human IgG1 Fc-HRP, clone HP6001	SouthernBiotech	Cat#9054-05; RRID:AB_2796627
Mouse anti-human IgG2 Fd-HRP, clone 31-7-4	SouthernBiotech	Cat# 9060-05; RRID:AB_2796633

**Bacterial and virus strains**		

GBS strains A909 (AlpC/CPS type Ia)	Tomas Areschoug (Department of Laboratory Medicine, Lund University, Sweden)	N/A
GBS strains BM110 (Rib/CPS type III)	Tomas Areschoug (Department of Laboratory Medicine, Lund University, Sweden)	N/A
GBS strains NCTC12906 (Alp1/CPS type Ia)	Tomas Areschoug (Department of Laboratory Medicine, Lund University, Sweden)	N/A
GBS strains NEM316 (Alp2/CPS type III)	Tomas Areschoug (Department of Laboratory Medicine, Lund University, Sweden)	N/A
GBS strain 14091 (Rib/CPS type III)	NCTC	Cat#14091
GBS strain 14092 (AlpC/CPS type Ib)	NCTC	Cat#14092
GBS strain 14093 (Rib/CPS type II)	NCTC	Cat#14093
GBS strain 14094 (Alp1/CPS type Ia)	NCTC	Cat#14094
GBS strain 14095 (Alp2/CPS type V)	NCTC	Cat#14095

**Biological samples**		

Baby rabbit complement (BRC; Pel-Freez, Arkansas)	Pel-freez Biologicals (Rogers, AR, USA)	Cat#31061

**Chemicals, peptides, and recombinant proteins**		

AlphaC-N protein	Bioneer A/S, Denmark	Specific order/Batch number: 5422
Rib-N protein	Bioneer A/S, Denmark	Specific order/Batch number: 5474
Alp1-N protein	Bioneer A/S, Denmark	Specific order/Batch number: 5352
Alp2/3-N protein	Bioneer A/S, Denmark	Specific order/Batch number: 5400
PBS 10X	Medicago AB	Cat#12-9423-5
Tween 20	Merck	Cat#P2287
NaCl	Sigma-Aldrich	Cat#S9888-10 KG
3,3,5,5-tetramethylbenzidine (TMB) dihydrochloride hydrate	Sigma-Aldrich	Cat#T8768
H_2_SO_4_	Merck	Cat#258105
N, N-Dimethylformamide	Sigma-Aldrich	Cat#227056-100 ML
Ethylenediaminetetraacetic acid (EDTA)	Sigma-Aldrich	Cat#E988
Bovine serum albumin (BSA)	Merck	Cat#A7906
Glycerol	Sigma-Aldrich	Cat#G7893-500 ML

**Experimental models: Cell lines**		

Human promyelocytic leukemia cell line HL-60	ATCC	Cat#CCL-240

**Software and algorithms**		

Prism7	GraphPad	https://www.graphpad.com/scientific-software/prism/

**Other**		

ELISA plate	Corning Costar, High Binding	Cat#3369
96-well Round bottom culture plates	Corning Costar, High Binding	Cat#3799
Gibco X10 RPMI 1640 medium (1X), liquid with L-glutamine	Gibco	Cat#21875091
Gibco™ Penicillin-Streptomycin (10,000 U/mL)	Gibco	Cat#15140-122
GlutaMax-1 (100X)	Gibco	Cat#35050038
Gelatin	Sigma-Aldrich	Cat#G9391-100G
Gibco™ HBSS (10X), no calcium, no magnesium, no phenol red	Gibco	Cat#14185-052
Gibco™ HBSS (10X), calcium, magnesium, no phenol red	Gibco	Cat#14065056
Bovine Serum (FetalClone I, for HL60 cells)	HyClone	Cat#SH30080.03
Fetal Bovine Serum (defined FBS, for OB)	HyClone	Cat#SH30070.03
Fetal Bovine Serum	Sigma-Aldrich	Cat#F7524
Todd-Hewitt-yeast extract (THY) broth	Substratavdelningen, Lund University	N/A
Todd-Hewitt-yeast extract (THY-agar)	Substratavdelningen, Lund University	N/A

### Resource availability

#### Lead contact

Further information and requests for resources and reagents should be directed to and will be fulfilled by the lead contact, Bengt Johansson-Lindbom (bengt.johansson_lindbom@med.lu.se).

#### Materials availability

All unique/stable reagents generated in this study are available from the lead contact with a completed Materials Transfer Agreement. Please direct resource and reagent requests to the lead contact specified above, Bengt Johansson-Lindbom.

### Experimental model and subject details

#### Human subjects

##### Recruitment of subjects

Subjects were healthy non-pregnant pre-menopausal female volunteers aged 18 to 40 with a body mass index (BMI) of 18.0–30.0 kg/m^2^ and weight 50–100 kg. All volunteers were fully informed about the content of the trial and provided signed informed consent following demonstration of understanding. Major exclusion criteria were significant medical conditions, including chronic, immunosuppressive, malignant, or gastrointestinal diseases or abnormalities. After a comprehensive screening evaluation, 60 subjects were enrolled and randomized to receive vaccine or placebo.

##### Study design

This was a randomized, double-blind, placebo-controlled, parallel group study conducted in a total of 60 healthy adult women who were not pregnant at the time of enrolment and were required to use an effective form of contraception throughout the active phase of the trial. Two cohorts of 30 subjects, randomized 4:1 active to placebo, were administered doses of 25 or 50 μg of each of GBS-NN and GBS-NN2 adsorbed to aluminum hydroxide (AlOH) as adjuvant at a dose of 0.5 mg Al^3+^. The vaccine was administered as an intramuscular injection, on two occasions, 28 days apart.

##### Production and administration of vaccine

The vaccine was manufactured and released according to Good Manufacturing Practice (GMP) requirements as four separate sterile components for reconstitution at the clinical pharmacy; GBS-NN, GBS-NN2, buffer and the AlOH adjuvant Alhydrogel® (Brenntag Denmark). The individual vaccine doses were assembled by diluting appropriate volumes of GBS-NN and of GBS-NN2 solutions with buffer and adding to a vial of Alhydrogel®. After thorough gentle mixing to allow adsorption of the antigen to the adjuvant, 0.5mL was withdrawn into a syringe for administration to subjects. Placebo was assembled by adding buffer to a vial of Alhydrogel®.

##### Primary and secondary objectives

The primary objective was to evaluate the safety and tolerability of the AlpN vaccine for 12 weeks after the first dose of vaccine, with a secondary safety objective to evaluate the long-term safety profile of the vaccine up to six months following the second dose.

The secondary immunological objective of the study was to evaluate IgG antibody responses induced by different vaccine doses at the primary endpoint, 12 weeks after the first dose, to select the optimum level to progress to Phase 2 development. The persistence of the serum IgG response to the vaccine up to six months following the second dose was also evaluated. Exploratory objectives included the evaluation of antibody responses to the individual Alp-N domains, the isotype profile, and the functional activity of the induced antibodies in an opsonophagocytic killing assay (OPKA).

##### Endpoints and definitions

The primary safety endpoints were local and systemic reactogenicity, adverse events (AEs), laboratory tests, urinalysis, vital signs, 12-lead electrocardiogram (ECG) parameters, and physical examination. The protocol-specified immunological endpoints, evaluated according to the primary Statistical Analysis Plan (SAP), were geometric mean concentration (GMC) of IgG specific for GBS-NN and GBS-NN2 in μg/mL, geometric mean fold increase in antibody concentration, and proportion of volunteers achieving antibody concentrations above specific thresholds (1, 2, 4, 8 μg/mL). The primary immunological outcomes were the values of these endpoints at day 85, 12 weeks after the administration of the first dose of vaccine and estimated to be close to the time of delivery if a pregnant woman was vaccinated at 26 and 30 weeks of gestation. Exploratory objectives were the evaluation of the IgG and IgA antibody responses to the individual N-domains (αC-N, Rib-N, Alp1-N, Alp2/3-N) and the ability of the sera to mediate killing of GBS strains expressing each of these allelic variants in the opsonophagocytic killing assay (OPkA).

##### Clinical safety monitoring

Safety of the GBS-NN/NN2 vaccine was assessed by evaluating local and systemic reactogenicity, AE’s, hematology, serum biochemistry, urinalysis, vital signs, 12-Lead ECG parameters and physical examination findings. Following completion of study day 85 (time of the primary endpoint), subjects were followed until 6 months after they received their second dose. In the follow-up period only serious adverse events and pregnancies were recorded.

### Method details

#### Quantification of vaccine-specific antibodies in sera from the study subjects

Cloning, production, and purification of recombinant Alp-N proteins has recently been described^29^ Concentrations of antibodies (IgG, IgG1, IgG2 and IgA) against the individual Alp-Ns, or the fusion proteins GBS-NN and GBS-NN2, were measured in quantitative ELISAs, using a human therapeutic subcutaneous immunoglobulin preparation as a calibrated reference standard (SCIG; Subcuvia, Baxter). Concentrations of IgG, IgG1, IgG2 and IgA against each individual antigen in the SCIG preparation had initially been determined based on equivalence in absorbance between a reference IgG, IgG1, IgG2 or IgA capture ELISA and the corresponding Alp-N specific ELISA performed in parallel under identical conditions,^37^^,^^38^ as previously described.^29^ The ELISA protocols were adapted from our previously published assays.^28^^,^^29^ Briefly, ELISA plates (Corning Costar, High Binding) were incubated overnight at 4°C with 100 μL recombinant αC-N, Rib-N, Alp1-N, Alp2/3-N, GBS-NN or GBS-NN2 per well, all diluted to 0.5 μg/mL in PBS. Pre- and post-vaccination sera were serially diluted in PBS containing 3% BSA (w/v) and 0.05% Tween 20 (v/v) (sample buffer) and added to the wells (100 μL/well). Calibrated SCIG was serially diluted in sample buffer and added to separate rows of wells on each plate (100 μL/well). Plates were incubated for 2 h at room temperature and after three washes in PBS with 0.05% Tween 20 (v/v) (wash buffer), the following titrated horseradish peroxidase (HRP)-conjugated detection antibodies, all diluted in sample buffer, were added to all wells (100 μL/well): Goat F(ab’)_2_ Anti-human IgG (SouthernBiotech, Cat. No. 2042-05), Mouse monoclonal anti-human IgG1 Fc (SouthernBiotech, clone HP6001, Cat. No. 9054-05), Mouse monoclonal Anti-Human IgG2 Fc (SouthernBiotech, clone 31-7-4, Cat. No. 9060-05) and Goat anti-human IgA Cross-Adsorbed (Invitogen, Cat. No. A18787). The plate was incubated with the HRP-conjugated reagents for 1 hour at room temperature. After three washes with wash buffer, HRP was detected using tetrametylbenzidine (TMB; Sigma T8768)-hydrogen peroxide (100 μL/well). Color reaction was stopped with 50 μL of 1 M sulfuric acid/well and the absorbance was read in SpectroStar photometer at 450 nm wavelength.

Assays measuring IgG against GBS-NN and GBS-NN2 were performed to GLP standards and data analyzed at Seirian Laboratories (Merthyr Tydfill, UK). Serum IgG concentrations specific for GBS-NN and GBS-NN2 were measured on days 1, 15, 29, 43, 57, 85 and 210. Responses to the different dose levels were analyzed by their geometric mean concentrations and the frequency of subjects who achieved pre-set concentration thresholds.

The responses to the four individual Alp-Ns were evaluated as exploratory objectives. Raw absorbance data were analyzed using four parameters logistic (4PL) fit function in Prism7 software (GraphPad). Concentrations of antibodies in serum samples were computed for all sample dilutions yielding results within the working range of the assay, using absorbance values of the calibrated SCIG dilutions as standard curves. For serum samples with concentration below lower level of quantification (LLOQ) of the assay, a concentration corresponding to 50% of the LLOQ was assigned.

#### Opsonophagocytic killing assay (OPkA)

The OPkA was adapted from the protocol described by Nahm and Burton,^30^ and performed as previously described.^29^ GBS strains A909 (αC; CPS Ia), BM110 (Rib; CPS III), NCTC12906 (Alp1; CPS Ia) and NEM316 (Alp2/; CPS III) were kindly provided by Tomas Areschoug (Department of Laboratory Medicine, Lund University, Sweden). The “GASTON” panel of target strains^31^ were obtained from The National Collection of Type Cultures (NCTC), UK Health Security Agency, UK and included NCTC strains 14092 (aC; CPS Ib), 14093 (aC; CPS II), 14091 (Rib; CPS III), 14094 (Alp1; CPS Ia) and 14095 (Alp2; CPS V).

### Quantification and statistical analysis

The primary objective of the study was a safety evaluation of the vaccine, and the sample size was considered adequate for this. With a total of 48 subjects receiving at least one dose of active vaccine in the presence of Alhydrogel®, the study would have a 95% probability of detecting AE’s occurring in 6.3% or more of the population. No power calculations were made based on immune response expectations. Dichotomous immunological results (e.g. numbers of subjects achieving threshold concentrations after different treatments) were analyzed using Fisher’s Exact Test. Quantitative immunological responses were logarithmically transformed to normalize the distributions and then analyzed by ANOVA to calculate geometric means and 95% confidence intervals. Direct comparisons between individual groups were analyzed by paired or unpaired t-tests of logarithmically transformed values. All statistical analyses were performed using GraphPad Prism version 9.1.0–9.1.2 for Mac, GraphPad Software, San Diego, California USA.

### Additional resources

Clinical trial number: NCT03807245.

## Data Availability

• All data reported in this paper (de-identified) will be shared by the lead contact upon request. • This paper does not report original code. • Any additional information required to reanalyze the data reported in this paper is available from the lead contact upon request.
