# Supplementary material for: Safety and immunogenicity of the group B streptococcus vaccine AlpN in a placebo-controlled double-blind phase 1 trial
Source: iScience. 2023 Feb 21;26(3):106261. doi: 10.1016/j.isci.2023.106261 (PMC10005905; doi:10.1016/j.isci.2023.106261)
Supplement: Document S1. Figure S1 and Tables S1–S3 [file mmc1.pdf]

iScience, Volume *26*

Supplemental information

Safety and immunogenicity of the group B *streptococcus* vaccine AlpN in a placebo-controlled double-blind phase 1 trial

Majela Gonzalez-Miro, Andrzej Pawlowski, Janne Lehtonen, Duojia Cao, Sara Larsson, Michael Darsley, Geoff Kitson, Per B. Fischer, and Bengt Johansson-Lindbom

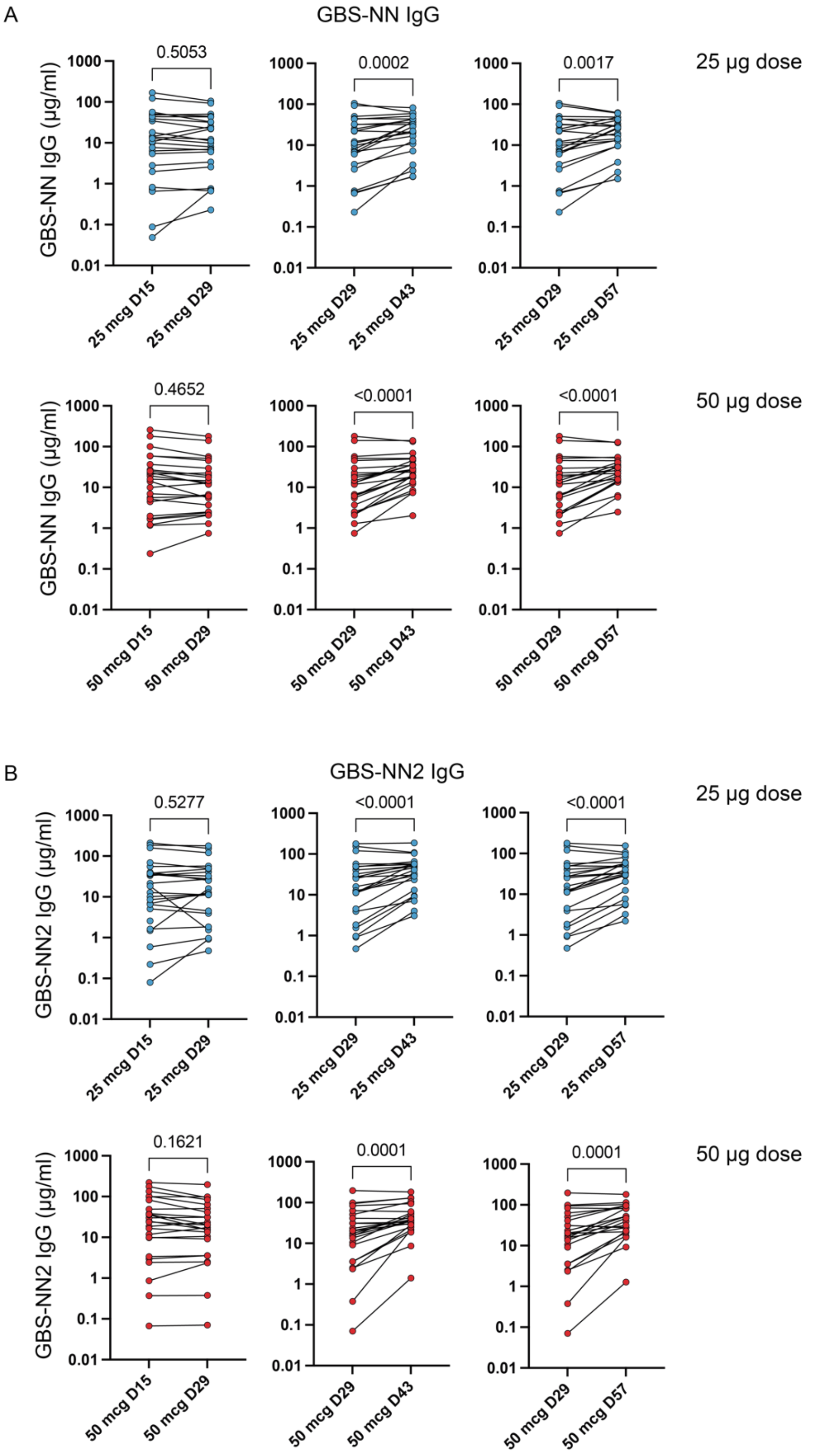

Figure S1. A second dose of the AlpN vaccine leads to significantly increased IgG levels. Individual pre- and post-boost levels of IgG against GBS-NN (A) and GBS-NN2 (B) for indicated dose levels. Paired t-test analyses for day 15 (2 weeks after primary) versus day 29 (4 weeks after primary), day 29 (4 weeks after primary) versus day 43 (2 weeks after boost), and day 29 (4 weeks after primary) versus day 57 (4 weeks after boost). Related to Figure 2.

Table S1. Subject Disposition. Related to Figure 1.

	2 x 25 µg + AlOH	2 x 50 µg + AlOH	AlOH (Placebo)
Randomised 1st dose	24	24	12
Received 2nd Dose	23*	24	12
Completed Day 85 visit	23*	24	12
Evaluable Per Protocol	23*	24	12
Evaluable for Immunogenicity	24	24	12
Completed Day 210 visit	23*	24	12

*One subject was withdrawn at request of the Sponsor for no longer being willing to comply with the study requirements and not replaced.

Table S2. A second dose of the AlpN vaccine results in increased percentages of subjects with specific IgG levels above arbitrarily set thresholds. Percent subjects in the 50-µg cohort reaching pre-defined thresholds in Alp-N specific IgG after the first and second dose of the vaccine. Related to Figure 4 and Table 4.

	**First dose (day 29)**			**Second dose (day 57)**		
**Alp-N**	>0.5 µg/ml	>1 µg/ml	>2 µg/ml	>0.5 µg/ml	>1 µg/ml	>2 µg/ml
**αC-N**	87%	78%	65%	100%	96%	91%
**Rib-N**	78%	70%	48%	96%	96%	83%
**Alp1-N**	91%	87%	74%	100%	96%	96%
**Alp2/3-N**	87%	78%	70%	100%	96%	96%

Table S3. AlpN vaccination results in enhanced opsonophagocytic killing of multiple GBS target strains and irrespective of the CPS serotype. Opsonophagocytic killing of the study versus the GASTON consortium GBS strain panels assessed with matched pooled pre- and post-vaccination sera from seven vaccinees. The two pooled sera were prepared using the same volume of individual pre- and post-vaccination serum samples. Related to Figure 4.

Alp-serotype	GBS strain[a]	Capsule type	OPkA titer Day 0[b]	OPkA titer Day 57[c]	ΔOPkA titer[d]
αC	**A909**	**Ia**	**777**	**5972**	**5195**
	NCTC 14092	Ib	1016	6852	5836
Rib	**BM110**	**III**	**1169**	**2407**	**1239**
	NCTC 14091	III	1112	2154	1042
	NCTC 14093	II	1957	5930	3973
Alp1	**NCTC 12096**	**Ia**	**1136**	**2500**	**1364**
	NCTC 14094	Ia	472	1441	969
Alp2	**NEM316**	**III**	**893**	**2961**	**2068**
	NCTC 14095	V	596	2330	1734

[a] Prototypic GBS strain used in study in bold, GASTON panels strains in regular text.

[b] Pooled day 0 pre-vaccination serum

[c] Pooled day 57 post-vaccination serum

[d] ΔOPkA titer; vaccine-induced OPkA titer = titer (day 57) - titer (day 0
